# Sparking interest in public health amongst future doctors to tackle our NHS sustainability crisis

**DOI:** 10.15694/mep.2019.000196.1

**Published:** 2019-11-04

**Authors:** Hannah Beetham, Duncan Cole, Rhiannon Phillips, Fiona Wood, Hannah Broughton, Cono Ariti, Behrooz Behbod

**Affiliations:** 1 Cardiff University

**Keywords:** curriculum design, public health, case-based learning, evidence-based medicine, healthcare sustainability

## Abstract

This article was migrated. The article was marked as recommended.

The new NHS ‘Long Term Plan’ has a particular focus on the public health issues of disease prevention and reducing health inequalities. However, medical students often perceive public health as abstract and irrelevant to clinical practice. We believe students need to be encouraged to appreciate wider public health issues and ultimately be able to apply clinical and public health tools to achieve change for patients and populations. Our aim is for all medical graduates to be able to apply public health and evidence-based principles to their chosen specialties. In the undergraduate curriculum, we hope to extend problem-, case-, and simulation-based learning into public health education, emphasise the context around statistics and epidemiology teaching, and make the teaching more relevant, tangible and enjoyable. Intercalated BSc students will gain an interdisciplinary perspective by joining Master of Public Health (MPH) students to learn about the prevention and control of disease and the promotion of health and wellbeing. They will also have opportunities to join the new *Health Intelligence Team (H.I.T.)*, on a voluntary reserve list to support Public Health Wales in the event of real investigations. We will evaluate these strategies, and we hope that medical educators worldwide will share their experience of innovative approaches to public health and evidence-based medicine teaching in response to this article, so that public health teaching may be improved.

## Introduction

We have a duty of care to all patients, ensuring optimal outcomes through evidence-based prevention, diagnosis and treatment. Our National Health Service (NHS) in the UK turned 70 recently, but is facing threats to its sustainability due to an ageing population and complex problems such as antimicrobial resistance, obesity, air pollution and socioeconomic inequality. One solution may be to emphasise a public health perspective in the education of tomorrow’s doctors, aligned with the new NHS ‘Long Term Plan’, which has a particular focus on disease prevention and reducing health inequalities ( NHS, 2019).

The General Medical Council (GMC) states that,

“ *Newly qualified doctors must be able to apply the principles, methods and knowledge of population health and the improvement of health and sustainable healthcare to medical practice.*” ( GMC, 2018)

Gillam *et al*. argue that ‘medical students in many countries graduate without feeling energized by their social purpose’. This could be due to full curricula and pedagogical issues that encourage the student perception that public health is ‘abstract’ and ‘irrelevant to clinical practice’ ( Gillam, Rodrigues and Myles, 2016). In contrast, we feel that many parallels can be drawn between clinical and public health medicine ( Table 1).

**Table 1.** Parallels drawn between clinical and public health medicine

Domain	Clinical methods	Public Health Medicine methods
**Symptoms**	**Patient history-taking**	**Asking the right questions to identify population health problems**
**Signs**	**Physical examination**	**Population studies** Health Needs Assessments, Health Equity Audits, Health Impact Assessments
**Investigations**	**Blood tests, cultures, urinalysis, etc.**	**Epidemiological research and statistical analyses**
**Diagnosis**	**Using gathered information to decide the potential causes of the problem that can be addressed**	**Triangulation of hard and soft intelligence to make interpretations and recommendations** Causes of the problem that can be addressed
**Action plan**	**Treatment** Medicine, surgery, etc.	**Public health intervention** Screening, immunisations, health promotion, community development, etc.
**Evaluation/monitoring**	**Follow-up and audit**	**Service evaluation**

To increase engagement with public health, we believe undergraduate education must stress the aims of, and context surrounding, epidemiology and statistics: where the most rewarding part of clinical medicine is reaching a diagnosis and treating the patient, the exciting part of public health medicine is addressing the health needs of a population through implementing evidence-based strategies.

Doctors in any clinical specialty who have a clear perception of their role in population health can help to improve services, ensure the effectiveness of interventions, and understand how best to allocate resources fairly ( Myles *et al.*, 2014). The effective application of evidence is of particular importance with the increasing amount of information available through the emergence of Big Data, artificial intelligence, and emerging technologies such as blockchain ( Behbod *et al.*, 2019), an open register offering several benefits including better data privacy, security and accuracy. This technology could help to enhance the interpretation of diagnostic investigations and disease prediction models, leading to more efficient healthcare delivery ( Kyriakoudes, Louca and Behbod, 2018).

## Our aims and strategy

We believe medical students should learn how to ask the right questions to identify the needs and pressure points within the health system, followed by identifying, critiquing and interpreting the information from a range of sources to translate knowledge into meaningful intelligence for action. In postgraduate training, junior doctors can build on their public health perspective by gaining competencies to generate a vision and strategy using systems thinking and leadership, and apply clinical and public health tools to achieve meaningful change ( Table 2).

**Table 2.** Using a public health approach to improve the health and care of patient

Use of systems thinking & leadership to translate information into action		
**Step 1: Assessment** *Where are we now?*	**Step 2: Vision** *Where do we want to be?*	**Step 3: Strategy** *How do we achieve our vision?*
Ask the right questions to identify the problems, needs and opportunities in a population.	Review evidence-based guidelines, consult stakeholders, and critically appraise the literature	Triangulate, interpret, critique and communicate findings*, and translate knowledge into effective actions for change. (*E.g. from statisticians, epidemiologists, policymakers, the public, subject-matter experts, economists, clinicians, etc.)

Additionally, we believe that medical school curricula should consider educational opportunities to develop the skills necessary for cross-industry partnerships. The problems we face require innovative and multi-sectoral solutions, facilitated by a public health perspective. Doctors will need to work not only within multidisciplinary teams within healthcare, but also with colleagues in other sectors. For example, doctors can work with: 1) qualitative and behavioural experts to improve the effectiveness of interventions; 2) architects, engineers and housing experts to reduce the risk of harmful environmental exposures that increase the risk of asthma, lung cancer or mesothelioma; 3) urban planners to improve access to cycle lanes, foot paths, and recreational areas; or 4) computer scientists to practice predictive, preventative and personalised medicine. This, however, is part of a long-term vision for medical education, and a potential development for the future, as it is not something we are implementing presently.

At Cardiff University, our intention is not to attract all medical students to specialise in public health, but rather for all medical graduates to apply public health and evidence-based principles to their chosen specialties. We aim to develop graduates that are able to identify and address problems within the health sector, whilst supporting the public health capacity of Wales.

We are planning to extend problem-, case-, and simulation-based learning into public health education in the undergraduate medical curriculum. For example, as part of the Evidence Based Practice module in year 3, we will focus the learning around student-identified health problems, guiding students through the process of developing the problem, gathering relevant information, critically evaluating the evidence and developing a plan for intervention. This will be supported by plenaries, eLearning and workshops. We hope that this will emphasise the context around statistics and epidemiology teaching, and make the teaching more relevant, tangible and enjoyable, as the students will have chosen an issue that is important to them.

Medical students that enrol on the Intercalated BSc in Population Medicine (an additional year of study between years three and four, or four and five) will gain an interdisciplinary perspective as mentioned above when joining Master of Public Health (MPH) students (many of whom come from a range of sectors) to learn about the prevention and control of communicable and non-communicable disease and the promotion of health and wellbeing. We will also be introducing the new *Health Intelligence Team (H.I.T.)*, where students will have the opportunity to serve on a voluntary reserve list to support Public Health Wales in the event of real-world investigations, such as disease outbreaks or environmental incidents.

## Next steps

We will evaluate our changes, and we hope that medical educators worldwide will also share their experience of innovative approaches to public health and evidence-based medicine teaching, reporting how these practices result in changes to medical students’ perceptions of public health.

## Take Home Messages

• A public health perspective may be needed to ensure the sustainability of the NHS
• The Cardiff University undergraduate curriculum will offer a new way of teaching which will guide students through a process of developing the public health problem, gathering relevant information, critically evaluating the evidence and developing a plan for intervention, in order to make public health teaching more enjoyable and engaging
• The intercalated BSc programme, will include interdisciplinary teaching, and encourage students to participate in the *Health Intelligence Team*, where students will have the opportunity to assist with real investigations

## Notes On Contributors

Hannah Beetham is a 3rd year medical student at Cardiff University School of Medicine and has interests in prevention of illness and promotion of health through population interventions and lifestyle change.

Duncan Cole is a Senior Lecturer in Medical Education and Deputy Director of the MBBCh, responsible for curriculum, at Cardiff University School of Medicine. He has interests in curriculum design, clinical reasoning, and technology-enhanced learning. He is also an Honorary Consultant in Medical Biochemistry and Metabolic Medicine in Cardiff and Vale UHB. ORDID: https://orcid.org/0000-0003-4690-8148

Rhiannon Phillips is a Health Psychologist, Lecturer in Health Psychology, and health services researcher. She has an interest in population health education and practice.

Fiona Wood is a Reader within the Division of Population Medicine at Cardiff University School of Medicine. She is a social scientist with an interest in qualitative research methods. ORCID: https://orcid.org/0000-0001-7397-4074

Hannah Broughton is a Lecturer in Public Health, and has interests in teaching epidemiology, and in lifestyle behaviours and health. ORCID: https://orcid.org/0000-0002-6231-9338

Cono Ariti is a Lecturer in Public Health and has an interest in teaching statistics in medicine and healthcare. ORCID: https://orcid.org/0000-0001-7615-0935

Behrooz Behbod is Director of the Master of Public Health Programme, and Evidence Based Medicine Lead, at the Centre for Medical Education, Cardiff University, and Consultant Epidemiologist, Public Health Wales. His interests include intersectoral public health education and training, role of technology (e.g. artificial intelligence, machine learning, blockchain) in health and education and planetary health. ORCID: https://orcid.org/0000-0002-5997-8367
