# Supplementary material for: Sparking interest in public health amongst future doctors to tackle our NHS sustainability crisis
Source: MedEdPublish (2016). 2019 Nov 4;8:196. [Version 1] doi: 10.15694/mep.2019.000196.1 (PMC10712495; doi:10.15694/mep.2019.000196.1)
Supplement: Peer review history

# Reviewer report: Reviewer response for version 1

**DOI:** 10.21956/mep.19931.r30231

**Published:** 2020-03-07

**Authors:** Ken Masters

**Affiliations:** 1 Sultan Qaboos University

This review has been migrated. The reviewer awarded 3 stars out of 5 An interesting piece on efforts to spark interest in public health amongst future doctors to tackle the UK NHS sustainability crisis. The paper identifies the broad problems, and then gives some ideas about what can be done.Although it does make intriguing reading, the paper lays out only very early ideas for a plan. The paper appears to fall midway between arguing an opinion and giving details of a strategy, and, as such, does not quite meet either requirement. The early opinion is not really developed in much detail, and neither is the strategy. I think it would have been more valuable for the paper to be divided into two papers: the first, as an opinion piece, that rigorously develops the reasoning and arguments supporting the need for such a plan. This will allow commentary on the opinion, and strengthen the base. Then, the second paper develops a detailed plan with, if possible, early experiments and their results.If the authors wish to retain the current structure for Version 2, then it would be a good idea to develop both parts, but that does run the risk that weaknesses in he early opinion will be built into the final strategy.

Reviewer Expertise:

NA

No decision status is available

# Reviewer report: Reviewer response for version 1

**DOI:** 10.21956/mep.19931.r30232

**Published:** 2019-11-04

**Authors:** Trevor Gibbs

**Affiliations:** 1 AMEE

This review has been migrated. The reviewer awarded 4 stars out of 5 An interesting paper that begins the journey to a very important topic. Quite clearly, the authors have a very focused vision in mind; I suppose the next step will be to put some "meat on the bones" of their curriculum purpose. I will look forward to reading further the development of this important topic, specifically how they intend to implement it into the already structured, and possibly over-populated curriculum, and its long term evaluation of effect.

Reviewer Expertise:

NA

No decision status is available
